# Satisfaction with end-of-life care and self-rated health among bereaved family members; a descriptive cross-sectional study in an intensive care context

**DOI:** 10.1186/s12904-026-02124-x

**Published:** 2026-04-30

**Authors:** Lena Palmryd, Tove Godskesen, Anette Alvariza, Åsa Rejnö

**Affiliations:** 1 Department of Health Care Sciences, Marie Cederschiöld University, Box 11189, Stockholm, 100 61 Sweden; 2 Perioperative Medicine and Intensive Care Function, Karolinska University Hospital, Solna, Stockholm, 171 76 Sweden; 3 Faculty of Nursing and Health Sciences, Nord University, Box 1490, Bodø, 8049 Norway; 4 Department of Public Health and Caring Sciences, Centre for Research Ethics & Bioethics, Uppsala University, BMC, Box 564, Uppsala, SE-751 22 Sweden; 5 Department of Research and Development/Palliative Care, Stockholms Sjukhem, Mariebergsgatan 22, Stockholm, 112 19 Sweden; 6 Department of Health Sciences, University West, Trollhättan, 461 86 Sweden; 7 Department of Medicine, Skaraborg Hospital Skövde, Skövde, 541 85 Sweden; 8 Skaraborg institute for Research and Development, Stationsgatan 3, Skövde, Sweden

**Keywords:** Cross-sectional, Decision-making, End-of-life, End-of-life care, Family, FS-ICU, Intensive care, Satisfaction, Self-rated health, Palliative care

## Abstract

**Background:**

In intensive care units, critically ill patients often require life-sustaining interventions. When these no longer benefit the patient, care is often transferred to end-of-life care. Family members may find themselves in a stressful situation, since they often act as proxies in decision-making processes, and support the patient nearing the end of life, while also coping with their own grief. The aim of this study is to investigate bereaved family members’ satisfaction with care, decision-making, the patients’ last hours of life, and their own self-rated health in end-of-life care in an intensive care setting.

**Method:**

A descriptive cross-sectional design with the questionnaire Family Satisfaction with Care in the Intensive care unit (FS-ICU 24) and its subscales FS-ICU Care and FS-ICU Decision-making was used: Bereaved family members from seven intensive care units in an urban region in Sweden participated. Data were analysed using descriptive and inferential statistics.

**Results:**

Bereaved family members (*n* = 141) reported overall satisfaction with end-of-life care, FS-ICU Total 77.6 (SD = 20.4); FS-ICU Care 79.5 (SD = 19.9), and FS-ICU Decision-making 74.8 (SD = 23.3). Dissatisfaction were reported by 12.3% (*n* = 17) of the family members with insufficient emotional support, 14.7% (*n* = 20) inconsistent information, and 17% (*n* = 24) limited control over patients’ care. Family members who rated their health as worse compared to a year ago, reported lower satisfaction in FS-ICU Decision-making (*p* = 0.05).

**Conclusion:**

This study shows an overall high satisfaction with end-of-life care in ICUs with summary scores in the upper quarter as measured by the FS-ICU 24. This study also uncovers a lack of attention to the individual needs of family members. Dissatisfaction stemmed from the items concerning insufficient emotional support, inconsistent information, and limited control over patients’ care. A markedly dissatisfied subgroup were also identified. These findings underscore the importance of paying attention to family members who express dissatisfaction with care and decision-making and items that received the lowest satisfaction scores. It is of paramount importance that when patients are cared for in ICU at the end of life, family members needs are identified and measures are taken to meet those needs, particularly for the most dissatisfied family members.

**Supplementary Information:**

The online version contains supplementary material available at 10.1186/s12904-026-02124-x.

## Background

Intensive care units (ICUs) provide care for critically ill patients who often require life-sustaining treatments [1]. When a patient’s condition reaches the point where life-sustaining care is no longer beneficial, the focus changes to end-of-life care [2]. In these situations, family members often experience personal distress caused by the impending loss of someone close to them and grief following the patient’s death [3].

In most cases, patients approaching end of life in ICU are unable to communicate their preferences, for example due to respiratory support from ventilators and the use of sedatives [4]. This situation often places family members in the role of proxies, where they are asked to contribute with what they believe the patients’ preferences would be [5]. This position entails understanding complex clinical information and their participation in the decision-making process [5–7]. Family members often express a desire to be involved in decisions [8], despite the burden of responsibility that often comes with acting as a proxy [9]. When doing so, family members may experience a sense of shared responsibility for the decisions made, even though the ultimate decision-making authority rests with physicians. Family members’ involvement in decision-making may be shaped by multiple factors, including the individual’s familial role and, importantly, the legal framework of the country. These frameworks vary significantly between nations, affecting both the extent of family participation and the authority granted to them in clinical decision-making. In Sweden, legal and organisational frameworks do not grant family members formal decision making authority regarding medical treatment; instead, their role is advisory, and physicians are responsible for making final decisions in the best interest of the patient [10]. Family members are nevertheless important sources of information about the patient’s values and preferences and are routinely involved in discussions. However, family members do not always experience that the support they are offered in decision-making is sufficient [11].

When delivering end-of-life care in the ICU palliative care, aspects such as relieveing suffering and promoting quality of life are essential for patients and their families [12]. International frameworks from the World Health Organisation (WHO) [13, 14] and the International Association for Hospice and Palliative Care (IAHPC) [15] emphasise the importance of addressing physical, psychological, social and existential needs at the end of life, including relief of distressing symptoms such as pain, dyspnea and anxiety [12, 16]. In the ICU, where dying often occurs in technologically complex environments, providing clear information, emotional support and opportunities for family presence are central aspects of high-quality end-of-life care. However, it is highlighted that family members may experience shortcomings in communication, emotional support and involvement in care, underscoring the need to better understand how end-of-life care is perceived in this setting [17].

Family members of critically ill patients in the ICU often experience substantial psychological strain, including anxiety, stress and shock [18]. Witnessing a close person in pain or other distressing symptoms may further heighten their stress [19, 20]. Depressive or post-traumatic stress symptoms may also occur during and after end-of-life care in ICUs, with elevated risk reported among women and younger family members [21]. However, another study found that the youngest family members reported the highest satisfaction with care, whereas male family members reported the lowest satisfaction [22].

Previous research shows that family members do not consistently report satisfaction with end-of-life care in ICUs, for example regarding the support offered to them [11, 23]. In Sweden, as in several other countries, family support in ICU care is challenged by staff shortages, high workload, limited structured follow-up for family members, and a lack of formalized grief support [24]. Studies have also shown that the health of family members can be negatively affected when a close person is cared for at the end of life in the ICU [3, 18, 19]. Structured communication may increase family members’ satisfaction and contribute to more realistic expectations of care [25]. Involving family members in intensive care rounds has also been associated with both higher satisfaction and improved mental health outcomes [26]. The self-rated health of family members of ICU patients has been explored by Ågren et al. [27], who found that health-promoting conversations with critical care nurses (CCNs), during and after the ICU stay can help reduce stress and support improved mental health.

However, fewer studies have examined satisfaction with care and decision-making in relation to family members self-rated health. Understanding this is important, as it may provide a more comprehensive understanding of family members’ experiences and guide interventions to improve both care quality and family well-being. More studies are therefore needed to better understand what family members’ perceive as satisfactory care and how their health is affected during and after the critical illness and death of a close person in ICU.

To date, the FS-ICU instrument has not been applied in Sweden. The Swedish specialised healthcare system, including intensive care, differs structurally and organisationally from that of neighbouring Nordic countries, with a more decentralised ICU care organisation, well-documented staffing shortages, lower wages, longer working hours for registered nurses, and the lowest ICU bed capacity per capita in Europe [28]. These conditions may shape family satisfaction in ways that warrant focused investigation. In addition, little is known about bereaved family members’ satisfaction with care related to their own well-being following the death of a close relative in the ICU. This study therefore aims to investigate bereaved family members’ satisfaction with care, decision-making, and the patients’ last hours of life, as well as their self-rated health, in end-of-life care in ICU settings.

## Method

### Design

The study used a descriptive cross-sectional design and is reported according to STROBE’s guidelines [29].

### Study context

This study encompassed seven of eight ICUs located in an urban region in Sweden. The eighth declined participation due to high workload. Of the participating ICUs, four offered general intensive care, while three were specialized in thoracic-, neuro- and central/trauma care. The ICUs varied in bed capacity, ranging from four to ten beds. Three of the units had predominantly single rooms, while the other units had a combination of single and multi-bed rooms. The care team responsible for patient care typically consisted of the patient’s attending physician from the ward unit, as well as anaesthesiologists, CCNs, assistant nurses from the ICUs, together with physiotherapists, counsellors and priests. CCNs had significant responsiblility for ensuring that family members and accompanying children felt welcome when visiting patients in the ICUs. The support also included facilitating communication between the family members and the healthcare team to ensure a clear understanding of the care goals. For CCNs, this often means supporting communication with family members and ensuring that they understand the meaning of the conversations. Counsellors and priests were also available to support family members. All ICUs adopted an open visiting policy, to the extent that the pandemic situation allowed, where family members were allowed to be present round the clock, and offered dedicated visiting rooms for family members.

### Procedure and participants

Approval for the units’ participation in the study was sought from the administrative head manager of each of the seven ICUs included. After approval, the head nurses of each ICU acted as liaisons and passed information to the first author about patients who died during the inclusion period. Information on bereaved family members’ names and telephone numbers were obtained by the first author from the deceased patients’ medical records. The inclusion criteria were: being a bereaved family member of an adult patient who was cared for and died in one of the included ICUs between July 1, 2021 and June 30, 2022. Following ethical recommendations from Sque et al. [30] regarding research with bereaved participants, the data collection began as earliest four months after the patient’s death. Data collection continued for a maximum of eight months after the patient’s death. Other inclusion criteria were: being over eighteen years when the patient died, proficient in Swedish, and being registered in the patient’s medical record as a primary contact person. The family members were contacted by telephone by the first author who provided information about the study. Family members who met the inclusion criteria and were willing to participate were provided with written information about the study along with the questionnaire. The questionnaire could be answered via a digital link or in paper format; family members received a link via email if choosing the digital option. For the paper format, the questionnaire was sent by post together with a pre-stamped envelope. The questionnaires were answered anonymously and all family members received a reminder after three weeks. Consent to participate was provided through returning the completed questionnaire.

**Fig. 1:**
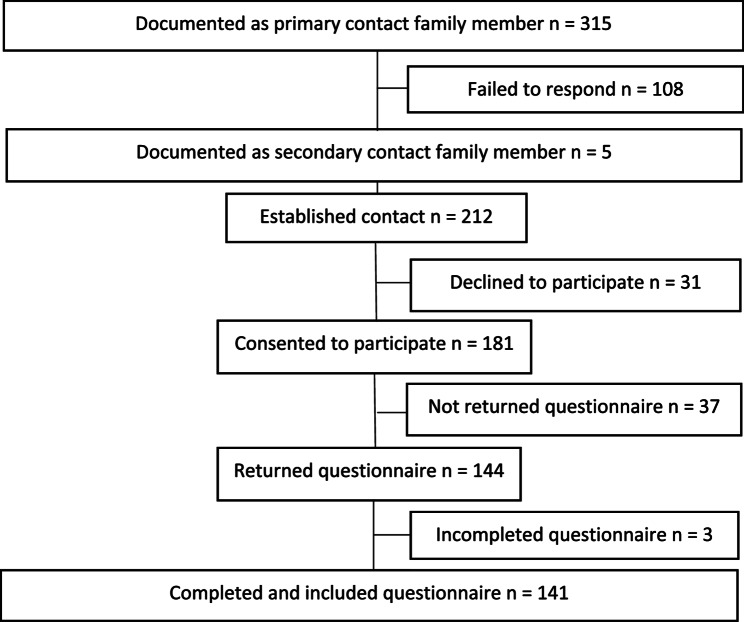
Flowchart showing the recruitment of participants

A total of 355 patients died in the included ICUs during the study’s inclusion period. Of these, 315 bereaved family members were documented as primary contact persons in the patients’ medical records. Five patients had two primary contacts listed. In cases where the first primary contact did not respond to outreach attempts, the secondary contact was approached. Three attempts were made to contact the family members, consisting of two phone calls and one text message. In five families, both the primary and the secondary contact persons responded to outreach efforts due to a delayed response from the primary contact. Finally, 144 (44%) family members returned the questionnaire, 140 of them digitally (Fig. 1).

### Questionnaire

The study-specific questionnaire consisted of: demographic questions about the family members such as sex, age, and reason for the deceased person’s stay in the ICU; Family Satisfaction with Care in the Intensive Care Unit, FS-ICU 24, three items from FS-ICU 24R, and two items from RAND-36. The questionnaire also included open-ended questions where participants could give supplementary answers in their own words (not used in present study).

The FS-ICU is available in several versions. The Swedish version of the validated English original version of the FS-ICU 24 [31] was selected for this study (Table 1). The FS-ICU 24 measures family members satisfaction with care and encompasses two subscales: FS-ICU/Care with items about satisfaction with care, and FS-ICU/Decision-Making (DM) with items about satisfaction with decision-making. For a list of all items included please see Supplemental file 1 and 2.

**Table 1:** Description of the instrument Family Satisfaction in the Intensive Care Unit, FS-ICU (24)

Created by	The Kingston General Hospital ICU Research Working Group in Canada by Heyland and Tranmer, 2001 [31].
Development	The original version consisted of 34 items and was subsequently refined and reduced to a 24 item version, FS-ICU 24, [34].
Construction	In the refinement of the FS-ICU 24, Wall et al. [34] found that the instrument consists of two subscales: *FS-ICU/Care*, which consists of 14 items related to satisfaction with patients care, interactions with healthcare professionals, and support tailored to individual needs; and *FS-ICU/DM*, which consists of ten items related to information provision, the availability of support, and involvement in the decision-making process. All response options follow a Likert scale, with values ranging from 1 to 5, where 1 indicates dissatisfaction and 5 indicates satisfaction. According to instructions [35], the values should be rescaled from 1–5 to 0-100, where 0 is worst and 100 is best. In terms of scoring, items that are answered as “not applicable” are considered “missing”. Questionnaires are excluded if responses to five or more items on the subscale FS-ICU/Care are missing, or if four or more items on the subscale FS-ICU/DM are missing, or if responses to eight or more of the total number of items are missing. The results are presented as summary scores for the total scale FS-ICU/Total, and the subscales FS-ICU/Care and FS-ICU/DM.
Validity and reliability	The FS-ICU 24 has undergone validation with Canadian and US populations and demonstrates strong reliability, as evidenced by Cronbach’s alpha coefficients of 0.92 for the FS-ICU Care subscale, 0.88 for the FS-ICU Decision Making subscale, and 0.94 for the FS-ICU Total [34]. The English version of the instrument by Wall et al. [34] has been assessed as having favourable psychometric properties [36].
Swedish version	FS-ICU 24 has been translated into Swedish by Söderström, Benzein, and Saverman [37]. The translation process involved linguistically appropriate adaptations of the response options. The Swedish version of FS-ICU 24 has not undergone validation.

Three items from the FS-ICU 24R, the most recent revised version of the instrument(not yet validated), were used to assess satisfaction with care during the patients’ last hours of life. Each item uses response options based on a five-point Likert scale. These items were included to capture aspects of end‑of‑life care that may be especially important for family members in the ICU context, where care at the end of life often is brief.The RAND-36 measures health and function in everyday life with a focus on physical, mental and social well-being [32]. Two self-rated items concerning general health were used, one about health today and one about health today compared to a year ago. The response options are presented on a five-point Likert scale ranging from “excellent” to “poor”. The Swedish version of RAND-36 is validated and has demonstrated good reliability with a Crohnbach’s alpha of 0.82 [33].

### Data analysis

Descriptive and inferential statistics were used to analyse the data. IBM SPSS Statistics version 27 and Microsoft 365 Excel were used for the data analysis. Descriptive statistics, such as mean value and standard deviation (SD), are presented for continuous data. Number, percentages, and minimum – maximum (min – max) are used to present categorical data. T-test was used to calculate p-values. A p-value of 0.05 was considered statistically significant.

Prior to the statistical analysis of FS-ICU 24, values were rescaled according to the scoring instructions [35], see Table 1. The rescaling means that a linear transformation was performed to standardize the response scale across all items [34]. The response option “not applicable” was classified as “missing” and these answers were exluded. 3 questionnaires were excluded in accordance with the instruction on how to handle questionnaires with missing items (see Table 1). Summary scores were calculated according to the scoring instruction, which means that first, the score, that is the sum of all items for each participant was divided by the number of items answered. The summary scores were then calculated as the mean value of all the scores.

A simplified scale was used to rank items with the highest proportion of satisfaction and dissatisfaction on the subscales of the FS-ICU 24. This involved combining the two response options for satisfaction (response options 1 and 2) and the two response options for dissatisfaction (response options 4 and 5), and the neutral response option (response option 3) was retained. Item 22 “Did you feel supported when decisions were made?” was excluded from the analysis due to a wording error in one of the response options, where the unintended inclusion of an extra word may have introduced ambiguity for participants.

Comparison of the summary score on FS-ICU 24 was made using t-test for the variables sex, age and health. In the analysis, the variable self rated health compared to a year ago was dichotomized as worse or same/better.

## Results

### Bereaved family members’ characteristics

Of the 141 family members, 90.0% (*n* = 126) were born in Sweden, 73.8% (*n* = 104) were employed, and 62.4% (*n* = 88) were women, who were younger than men (mean age 51.5 vs. 53.8 years). (Table 2).

**Table 2:** Characteristics of family members, shown by sex and in total (*n* = 141)

Family members’ characteristics, *n* = 141	Women	Men	Total
Age years; Mean, (SD) [min – max]	51.5 (14.6) [20 – 83]	53.8 (15.4) [25 - 80]	52.4 (14.9) [20 –83]
Sex; *n* (%)	88 (62.4)	53 (37.6)	141 (100)
Education; *n* (%)			
Elementary school	7 (8.0)	5 (9.4)	12 (8.5)
High school	30 (34.0)	30 (56.6)	60 (42.6)
College/University	51 (58.0)	18 (34.0)	69 (48.9)
Employment; *n* (%)			
Employed	67 (76.1)	37 (69.8)	104 (73.8)
Retired	16 (18.2)	12 (22.6)	28 (19.9)
Other	5 (5.7)	4 (7.6)	9 (6.3)
Relation to the patient; *n* (%)			
Husband/wife/partner	30 (34.1)	20 (37.7)	50 (35.5)
Parent	19 (21.6)	8 (15.1)	27 (19.1)
Adult child	30 (34.1)	17 (32.1)	47 (33.3)
Sibling	2 (2.2)	6 (11.3)	8 (5.7)
Other	7 (8.0)	2 (3.8)	9 (6.4)
Country of birth; *n* (%)			
Sweden	76 (86.4)	50 (96.2)	126 (90.0)
Other countries	12 (13.6)	2 (3.8)	14 (10.0)
Reason for patient’s care; *n* (%)			
Thoracic surgery/heart or lung disease	17 (19.3)	13 (24.5)	30 (21.3)
Neurosurgery/stroke	16 (18.2)	7 (13.2)	23 (16.3)
Covid-19	13 (14.8)	10 (18.9)	23 (16.3)
Accident/trauma	9 (10.2)	5 (9.4)	14 (9.9)
Infection	9 (10.2)	4 (7.5)	13 (9.2)
Unspecified illness	24 (27.3)	14 (26.4)	38 (27.0)

### Overall satisfaction with care according to FS-ICU 24

Bereaved family members reported high overall satisfaction with end-of-life care with an FS-ICU/Total summary score of 77.6, and with FS-ICU/Care rated higher than FS-ICU/DM (Table 3). However, scores varied widely, ranging from 6.82 to 100, and 10 family members reported summary scores below 40 (data not shown).

**Table 3:** Satisfaction with care in the ICU reported on FS-ICU/Care, FS-ICU/DM, and FS-ICU/Total

Subscales and Total scale	Summary scores (0–100)
FS-ICU/Care	79.5 (SD = 19.9)
FS-ICU/DM	74.8 (SD = 23.3)
FS-ICU/Total	77.6 (SD = 20.4)

### Bereaved family members’ satisfaction with care

**Fig. 2:**
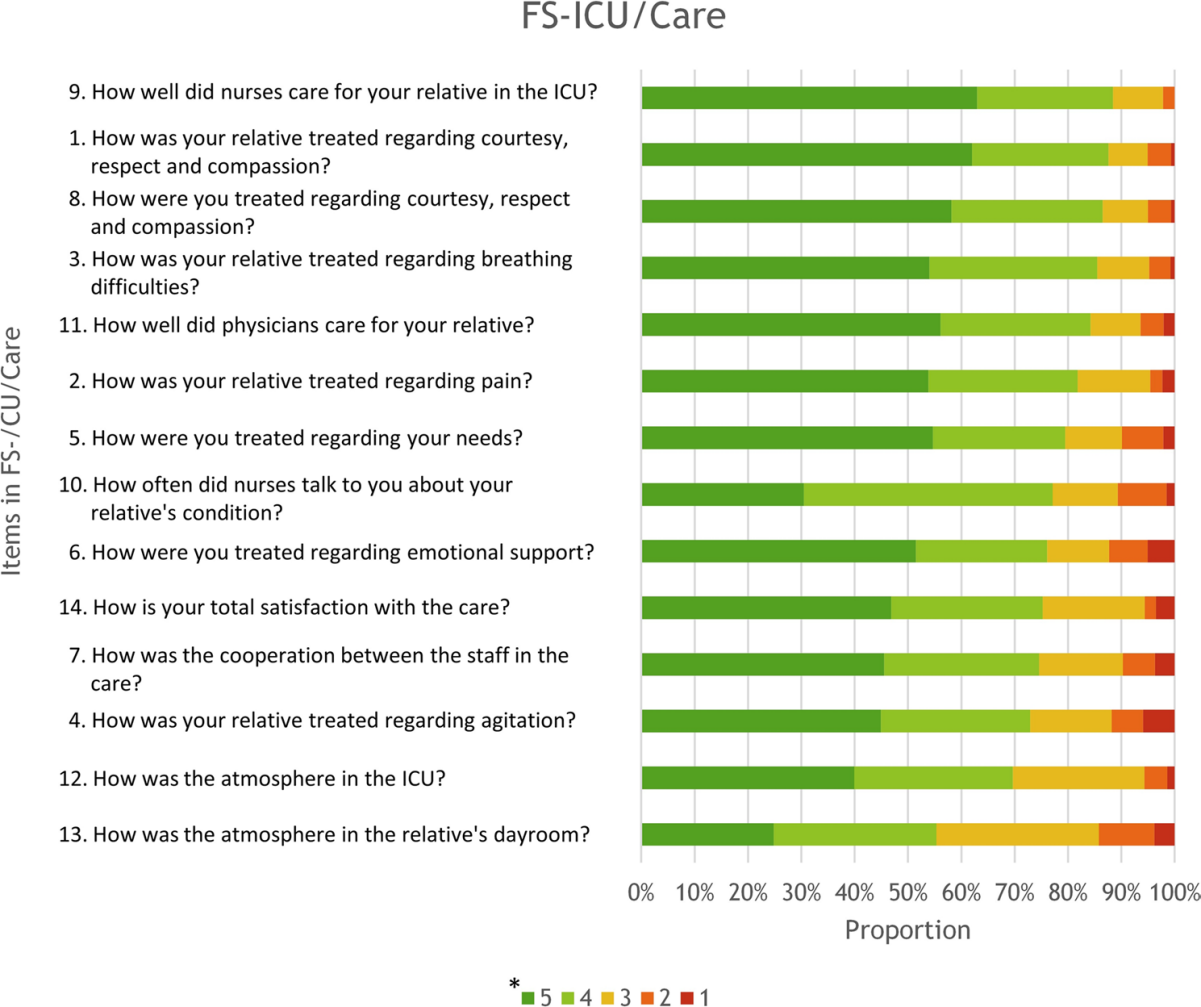
FS-ICU/Care showing items and response frequency *Likert scale responses ranging from 5 (most positive options = satisfaction) to 1 (most negative options = dissatisfaction). The two green colours in the staple chart represent the positive options, the yellow colour represents the neutral option, and the two orange/red colours represent the negative options

The three items indicating the highest proportion of satisfaction with FS-ICU/Care when using the simplified scale were items 9, 1 and 8 (Fig. 2 and supplemental file 1). Overall 88.4% (*n* = 122) were satisfied with the nurses’ care of patients (item 9), 87.6% with the healthcare staff’s courtesy, respect, and compassion for patients (item 1), and 86.6% with the healthcare staff’s courtesy, respect, and compassion for family members (item 8).

The three items indicating the highest proportion of dissatisfaction on FS-ICU/Care when using the simplified scale were items 13, 6, and 4. Overall, 14.2% were dissatisfied with the atmosphere in the family members’ dayroom (item 13), 12.3% with the emotional support regarding their needs (item 6), and 11.8% with symptom management when assessing and treating patients’ agitation (item 4).

### Bereaved family members’ satisfaction with decision-making

**Fig. 3:**
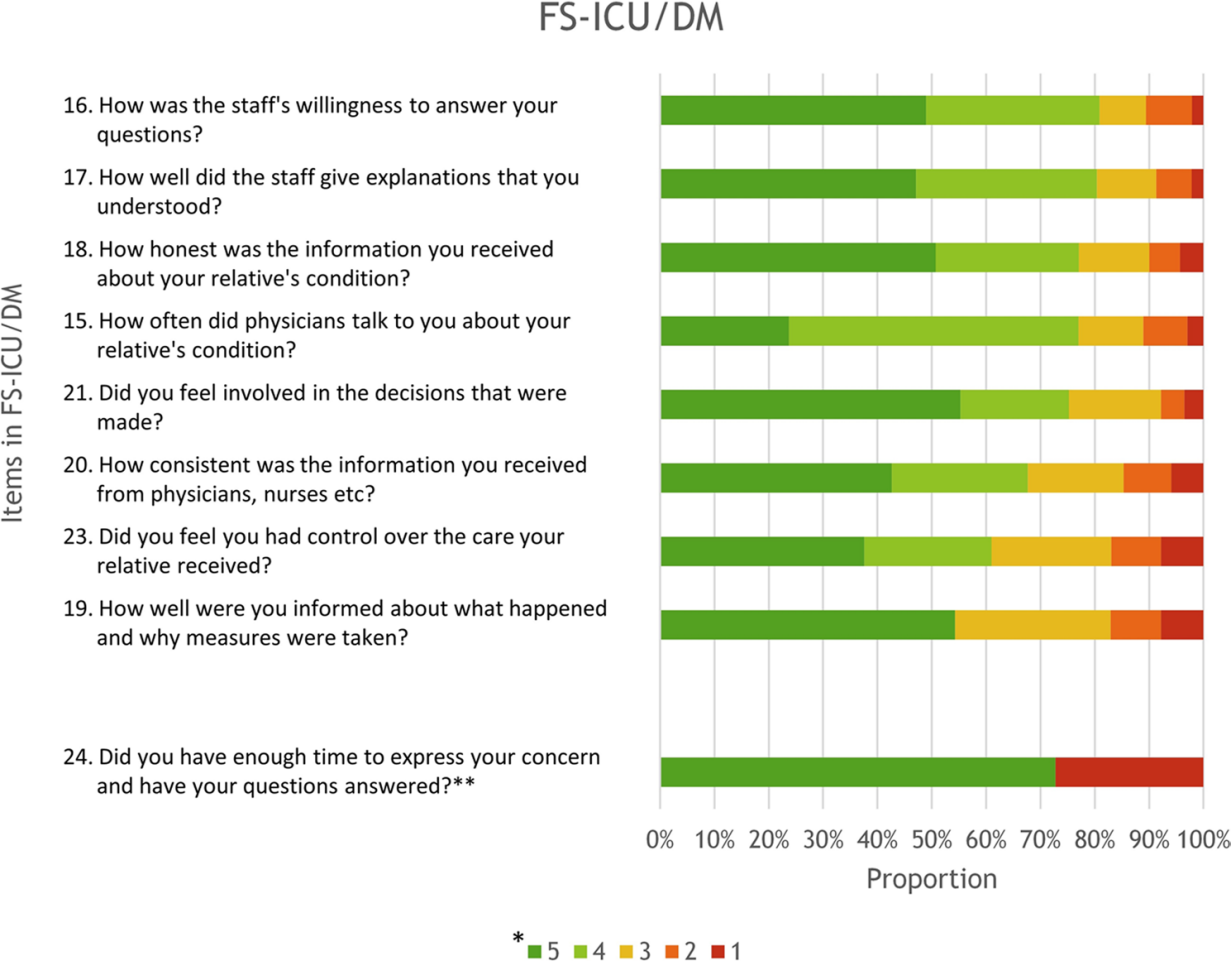
FS-ICU/DM showing items and response frequency *Likert scale responses ranging from 5 (most positive options = satisfaction) to 1 (most negative options = dissatisfaction). The two green colours in the staple chart represent the positive options, the yellow colour represents the neutral option, and the two orange/red colours represent the negative options **Item 24 is an item with dichotomized response options; “yes” visualized in green colour, and “no” visualized in red colour

The three items indicating the highest proportion of satisfaction on FS-ICU/DM when using the simplified scale were items 16, 17 and 18 (Fig. 3 and supplemental file 2). Overall, 80.8% were satisfied with the healthcare staff’s willingness to answer questions (item 16), 80.4% with explanations that were easy to understand (item 17), and 77.1% with the honest information provided about the patient’s condition (item 18).

The three items indicating the highest proportion of dissatisfaction on FS-ICU/DM when using the simplified scale were items 19, 23 and 20. Overall, 17.1% were dissatisfied with the information provided about what had happened to the patient and why measures were taken (item 19), 17% with the control they had over the patient’s care (item 23), and 14.7% reported that the information provided by physicians, nurses etc. was inconsistent (item 20).

### Bereaved family members’ satisfaction with patients’ last hours of life from the FS-ICU (24R)

The majority, 86.2%, reported that they experienced patients’ lives as neither unnecessarily prolonged nor shortened (item 25) (Table 4). However, 7.3% felt that the patients had a very difficult time during their last hours of life (item 26), while 2.9% felt themselves completely abandoned by the healthcare staff (item 27).

**Table 4:** Satisfaction with patients’ last hours of life from FS-ICU (24R)

Item	*n*	*n* (%)	*n* (%)	*n* (%)	*n* (%)	*n* (%)
25. Which of the following best describes your views? *I feel that my relative’s life was*		**prolonged unnecessarily**	**prolonged somewhat unnecessarily**	**neither prolonged nor shortened unnecessarily**	**shortened somewhat unnecessarily**	**shortened unnecessarily**
	138	2 (1.4)	3 (2.2)	119 (86.2)	9 (6.5)	5 (3.6)
26. During the last hours of your relative’s life, which of the following best describes your views? *I feel that my relative*		**had a very difficult time**	**had a hard time**	**was mostly safe and well**	**was very safe and well**	**was completely safe and well**
	137	10 (7.3)	11 (8.0)	37 (27.0)	28 (20.4)	51 (37.2)
27. During the last hours of your relative’s life, which of the following best describes your views? *I felt*		**completely abandoned by the staff**	**somewhat abandoned by the staff**	**neither abandoned nor supported by the staff**	**supported by the staff**	**very supported by the staff**
	139	4 (2.9)	4 (2.9)	14 (10.1)	46 (33.1)	71 (51.1)

### Bereaved family members’ self-rated health

Women and men reported comparable ratings of good general health (Table 5). A small percentage, 4.5% of the women and 1.9% of the men, rated their health as poor. Overall, about half of the family members perceived their health to be about the same today compared to one year ago. No statistically significant differences were observed between the sexes. However, more women than men rated their health as much worse today compared to a year ago (14.8% vs. 3.8%).

**Table 5:** Family members’ self-rated health, shown by sex and in total (*n* = 141)

Family members’ self-rated health, *n* = 141	Women	Men	Total
Generally: score (SD)			
- excellent	15 (17.0)	8 (15.1)	23 (16.3)
- very good	21 (23.9)	17 (32.1)	38 (27.0)
- good	32 (36.4)	19 (35.8)	51 (36.2)
- fairly good	16 (18.2)	8 (15.1)	24 (17.0)
- poor	4 (4.5)	1 (1.9)	5 (3.5)
Today, compared to a year ago: score (SD)			
- much better	4 (4.5)	2 (3.8)	6 (4.3)
- somewhat better	10 (11.4)	5 (9.4)	15 (10.6)
- same	42 (47.7)	29 (54.7)	71 (50.4)
- somewhat worse	19 (21.6)	15 (28.3)	34 (24.1)
- much worse	13 (14.8)	2 (3.8)	15 (10.6)

### Bereaved family members’ summary scores on FS-ICU/Care, FS-ICU/DM and FS-ICU/Total related to sex, age and self-rated health

When comparing summary scores on FS-ICU 24, related to sex and age respectively, small differences were seen but these were not statistically significant. A statistically significant difference (*p* = 0.05) was found in FS-ICU/DM between family members who rated their health as worse (mean 69.4) and those rating it same/better (mean 77.5) compared to a year ago (Table 6).

**Table 6:** Summary scores on FS-ICU/Care, FS-ICU/DM and FS-ICU/Total based on sex, age and self-rated health

	FS-ICU/Care	FS-ICU/DM	FS-ICU/Total
Sex, score (SD)			
-Women	79.4 (21.2)	75.0 (25.0)	77.7 (21.7)
-Men	79.9 (18.1)	74.2 (20.7)	77.5 (18.5)
-*p*-value, (sig. between groups)	0.904	0.840	0.948
Age, score (SD)			
-Under the age of 65 years	80.4 (19.9)	75.4 (24.0)	78.3 (20.7)
-65 years or older	77.4 (20.8)	72.8 (21.7)	75.6 (20.2)
-*p*-value (sig. between groups)	0.466	0.589	0.515
Health, compared to a year ago, score			
(SD)			
-Worse	76.2 (21.4)	69.4 (26.4)	73.3 (22.5)
-Same/better	81.4 (19.1)	77.5 (21.3)	79.9 (19.1)
-*p*-value (sig. between groups)	0.138	0.050	0.072

## Discussion

To the best of our knowledge, this is the first study in a Swedish context to examine bereaved family members’ satisfaction with care, decision-making, experiences of patients’ last hours of life, and the first to explore this in relation to their self-rated health. The summary scores in FS-ICU/Total are in the upper quarter of the scale, indicating high overall satisfaction with end-of-life care in the ICUs. The results show a higher satisfaction for the subscale FS-ICU/Care than the subscale FS-ICU/DM. However, items from FS-ICU 24R show that patients were perceived to have a difficult time during their last hours of life. Finally, those family members who reported deterioration in their own health compared to a year ago also reported a lower satisfaction with care and decision-making than those who reported the same or better health.

### FS-ICU/Total

The FS-ICU/Total summary score was 77.6, indicating that bereaved family members were generally satisfied with the end-of-life care provided in the ICUs. It may be assumed that satisfaction would differ depending on whether the patient survived or died. However, previous studies report mixed findings, with both higher and lower satisfaction among families of survivors. Our findings are consistent with other studies using the FS-ICU 24, such as Osborn et al. (10) (FS-ICU/Total 76.6) in bereaved family members, and Min et al. [38] (FS-ICU/Total 75.4) and Schwarzkopf et al. [39] (FS-ICU/Total 78.3) in which family members of surviving patients were also included. A somewhat lower summary score was observed by Haave et al. [22] (FS-ICU/Total 70.4) including family members of both surviving and non-surviving patients. As our study included only family members of patients who died, survival status cannot explain variation in satisfaction within our sample.

Although the overall FS-ICU/Total mean score in present study indicates general satisfaction, the wide variation in responses reveals a distinctly dissatisfied subgroup. This suggest that current practices do not fully meet the needs of all family members and highlights the importance of identifying and supporting those whose experiences diverge significantly from the overall pattern. In general, variation in reported satisfaction could theoretically arise from several factors, such as the emotional state of family members, the time interval between the onset of illness, ICU admission, and death, or practical aspects such as the time of day they arrived at the unit. The closeness of the relationship to the deceased may also influence how the care experience is perceived. To our knowledge, no previous FS-ICU study has reported on low-scoring subgroups. However, this does not preclude its existence, as a similar subgroup may well have been present but not noticed or reported. Yet, because the range of summary scores is rarely presented in prior publications, readers cannot determine whether comparable patterns were present in those samples.

### Comparison between FS-ICU/Care and FS-ICU/DM

Of the two subscales, FS-ICU/Care had a higher summary score (79.5) than FS-ICU/DM (74.8). This pattern is consistent with findings from Schwarzkopf et al. [39], Haave et al. [22], and Frivold et al. [40], suggesting that families in several European countries are generally more satisfied with patient care than the decision-making process. In contrast, studies from Asia have resported the opposite pattern, with higher satisfaction with decision-making than with care [38, 41]. Two possible explanations were suggested; low reimbursement for critical care limiting hospitals’ will to invest in ICU services and medical legislation that strenghtens family members’ right to information. Togheter, these regional differences underscore the the importance of context when interpreting family members satisfaction data.

### The FS-ICU/Care subscale

The FS-ICU/Care summary score of 79.5 in the study is is comparable to that reported by Osborn et al. [11] (77.7) in bereaved family members, and aligns with or slightly higher than reported by Schwarzkopf et el [39]. (78.6), Haave et al. [22] (74.1), and Frivold et al. [40] (73.0) in samples including both surviving and non-surviving patients.

In the study, the item on FS-ICU/Care indicating the highest satisfaction, was how well the nurses cared for the patient (item 9), followed by satisfaction of family members regarding courtesy, respect and compassion towards the relative (item 1), and towards family members (item 8) using the simplified scale. These findings are consistent with other studies reporting high satisfaction with care [22, 39, 42]. For instance, the study by Jensen et al. [42] who found that a significant majority of family members were satisfied with the courtesy, respect and compassion shown towards patients by 97.3%, and towards family members by 91.9% when applying our simplified scale to their results. Furthermore, a relationship has been noted between family members’ satisfaction with end-of-life care in ICU and family member characteristics; femalefamily decision-makers reported higher levels of satisfaction than male family decision-makers [41]. The relatively high satisfaction levels observed in our study may therefore partly reflect predominance of women among respondents.

In the present study 14.2% of the family members reported highest dissatisfaction with the atmosphere in the waitingroom (item 13) on FS-ICU/Care which is line with several previous studies [22, 38, 39, 43]. Newey et al. [43] referred to the waiting room feeling unwelcoming and cold. However, it may be that dissatisfaction with the waiting room reflects not its physical features, but rather the distress associated with being separated from the patient. If so, efforts targeting the waiting room atmosphere may not achieve its intended effect. The atmosphere in the ICUs (item 12) also received scores of dissatisfaction at the lower end by 5.7% of the family members in our study. Osborn et al. [11] showed that the atmosphere in ICUs is of importance in care and indicated potential avenues for improvement.

Furthermore, in present study, emotional support (item 6) was the second most common item of dissatisfaction reported by 12.3% of family members. This can be compared to Min et al. (36) where 8.5% were dissatisfied with emotional support, and Jensen et al. [44] who found a rate of 4.7%. These results demonstrate a consistent trend when applying the simplified scale used in the present study to earlier work [38, 44]. Emotional support has been shown to shape to family members overall experience of the care, particularly in end-of-life situations involving decisions about withdrawing and withholding life-sustaining treatments [23]. Carlson et al. [45] likewise reported that the emotional support provided is often insufficient given the overwhelming emotions family members face while managing the complexities of the patient’s condition. Osborn et al. [11] also highlighted dissatisfaction with emotional support and underscored the need for improved support. Taken together these findings reinforce the relevance of our study’s results and indicate that addressing emotional support should be a key focus for improving the experiences of bereaved family members in the ICU.

### The FS-ICU/DM subscale

In present study, the summary score on FS-ICU/DM was 74.8, in line with Osborn et al. [11] 75.2) and Chiang et al. [41] (76.1) for bereaved family members. Comparing with previous studies, the present study’s score aligns with Schwarzkopf et al. [39] (77.8), whereas the score in Haave et al. [22] (65.2) reported a lower score; however, both studies included family members of surviving and non-surviving patients. This indicates that the outcome of the patient’s intensive care stay, whether survival or non-survival, does not appear to determine how family members evaluate their satisfaction with the decision-making process.

On the FS-ICU/DM, 80.8% of family members reported satisfaction with the healthcare staff’s willingness to answer questions (item 16). When applying the simplified scale present study reports a lower summary score than that found by da Rosa Viana et al. [46] (86.1%), but a higher score than that reported by Frivold et al. [40] (71.9%). Both of these studies included family members of surviving as well as non-surviving patients. Further, satisfaction with the ability of the healthcare staff to give explanations that were understandable (item 17) was reported by family members with a lower summary score in our study (80.4%) compared to da Rosa Viana et al. [46] (88.7%), and a higher score compared to Frivold et al. [40] (62.9%), when applying the simplified scale. As stated by Chiang et al. [41] female family members tend to report higher satisfaction with decision-making than male family members. In present study, 62.4% of the family members were women. In comparison da Rosa Viana et al. (37) reported a higher proportion of women (79.5%) and reported higher summary scores, whereas Frivold et al. [40] reported a lower proportion of women (59.0%) and reported lower scores. Taken together these findings may reinforce the pattern suggested by Chiang et al. [41] namely that the proportion of women in the sample may influence the FS-ICU DM subscale scores.

On the FS-ICU/DM, dissatisfaction was reported regarding both information provided about what happened and why measures were taken (item 19) and concerning control over patients’ care (item 23). This sense of limited control is echoed in other studies using FS-ICU 24 [11, 39]. For example, Osborn et al. [11] suggested that insufficient communication between healthcare staff and family members may contribute to perceptions of inadequate control over the patient’s care. Schwarzkopf et al. [39], on the other hand noted that limited involvement in the patient’s care can lead family members to feel sidelined and assume that information is being withheld. In the light of this, concerns about communication and involvement appear central to how families assess decision-making. The present study demonstrates that these issues remain highly relevant in the Swedish ICU context and emphasize the need to for targeted efforts such as structured communication routines, clearer information pathways, and opportunities for meaningful family involvement.

### The questions from FS-ICU 24R

During the last hours of life, 7.3% of the family members in present study perceived that the patient had a were a very difficult time (item 26), indicating that they felt the patient’s suffering was not adequately relieved. In the context of Swedish healthcare, it has been shown that although pain control is a central focus in end-of-life care, pain remains a persistent and challenging symptom across a range of diagnoses [47]. Obstructed airways and respiratory distress, manifested as noisy breathing, can also be a significant source of concern for family members [48]. Among patients dying in ICUs, nurses have emphasized the importance of supporting family members well-being [48]. Our findings underscore that despite overall high satisfaction, a subgroup of family members still perceive substantial suffering during the patient’s final hours, highlighting the need to ensure that symptom relief and communication about symptom management are consistently addressed.

### Family members’ perceived health and relationship to satisfaction on the FS-ICU

A perceived deterioration in health status compared to one year previously was reported by 34.7% of family members in the present study. Importantly, this study reports a statistically significant association between poorer self-rated health and and lower satisfaction scores across both subscales and the total FS-ICU score. A previous review has shown that lack of emotional support, negatively affects the FS-ICU/Care score [23], a pattern also observed in present study. Da Rosa Viana et al. [46] similarly found that three months after a patients death in the ICU, family members reported reduced quality of life and an increased risk of developing anxiety and depression, compared with family members of surviving patients. These findings indicate that family members’ own health and emotional well-being are closely linked to how they evaluate the care and decision-making processes, and our results suggest that this relationship remains highly relevant within the Swedish ICU context.

### Strengths and limitations

An important strength of this study is the broad inclusion of family members from ICUs across an urban region, with participation from seven out of eight units representing different specialties. As the study period coincided with the COVID-19 pandemic, changes in ICU visiting policies may have influenced family members’ experiences, although this was not systematically assessed. Additionally, the cross-sectional design enabled the capture of experiences from a diverse group of grieving family members within a clearly defined time frame. Another strength is that the original English version of FS-ICU 24 has undergone validation, is reliability tested and has been shown to have good psychometric properties [34]. However, the Swedish instrument has not undergone similar validation or reliability testing. The FS-ICU instrument’s primary strength lies in its usefulness as a tool for monitoring the quality of clinical care within one’s own setting.

Caution is advised when interpreting the FS-ICU results across different studies due to ambiguities in its instructions for calculating summary scores for the subscales and the total scale [35]. We understand the instructions as if summary score is to be calculated as the total sum of all response options divided by the number of responses. Due to the correlational design, no causal inferences can be drawn from the observed associations between variables. Furthermore, variations in question phrasing and response options across different FS-ICU language versions necessitate careful comparisons between studies, since these variations in wordings may affect the instrument’s equivalence with the orginal English version of the FS-ICU 24. Several previous studies have also reported the FS-ICU instrument to have a clear ceiling effect [39, 42, 44]. This limits the ability to detect variation among family members with high satisfaction and reduces the instrument’s capacity to capture meaningful changes or identify specific areas needing improvement. Finally, we were primarily interested in investigating which items family members were satisfied or dissatisfied with, rather than the degree of satisfaction itself. The presence of a ceiling effect further supports this focus, as the FS-ICU instrument provides limited differentiation among highly satisfied respondents. Moreover, as individuals vary in their tendency to select extreme response options, shaped by both question characteristics and respondents traits, the decision to simplify the scale for one sub-analysis is justified.

Another limitation of this study is that we did not conduct a separate validation of the three items utilized from the FS-ICU 24R, the most recent revised version of the FS-ICU questionnaire. The FS‑ICU 24R is based on the original English FS‑ICU 24 version, which has demonstrated good psychometric properties, including reliability and validity, across multiple settings. As the revised version retains the same items and scoring structure, the existing validation evidence for the original instrument is considered applicable to the FS‑ICU 24R. This limitation should therefore be taken into account when interpreting the findings.

The study has a descriptive design, and therefore no sample size calculation was conducted. However, a few statistical hypothesis tests were performed. To confirm the statistically significant finding regarding self-rated health compared to one year ago, larger studies are warranted. A further limitation concerns the low response rate (44%), which could be attributed to a number of factors. Firstly, the study was conducted four months after family members had experienced the death of a next of kin. It is possible that family members experienced heightened emotional distress during this critical period, which might have resulted in them declining to participate. The low participation rate is also a limitation in terms of the study’s power, since it precludes the opportunity to demonstrate differences that exist but which, in this material, did not reach statistical significance. Secondly, while the majority of family members in the present study reported satisfaction with the end-of-life care provided, it is possible that those who were dissatisfied chose not to participate. Thirdly, the fact that the questionnaire was only provided in Swedish may have contributed to the lower number of participants with a background other than Swedish.

## Conclusion

This study shows an overall high satisfaction with end-of-life care in ICUs, with FS-ICU 24 summary scores in the upper quartil. However, the wide variation in scores and the presence of a smaller subgroup reporting markedly low satisfaction highlights the need to better identify vulnerable family members and adapt care strategies to their individual needs.

Dissatisfaction was mainly related to insufficient emotional support, inconsistent information, limited control over the patient’s care, and the distress observed during the patient’s last hours of life, indicating that family members’ needs were not sufficiently addressed. Shortcommings in the FS-ICU instrument also became apparent, underscoring the need for a revision and revalidation if it is to serve as a robust research tool, although its use for local quality improvement purposes appear less problematic.

While no causal relationship can be inferred between lower satisfaction and poorer self-related health the findings underscore the importance of early identification of dissatisfied family members and targeted attention to low scoring care domains. These domains highlight specific aspects of care where improvments may warrant further quality improvement efforts. Systematically identifying and addressing family members’ needs in the end-of-life ICU care may improve satisfactionn and may also help reduce the risk of adverse long-term psychological outcomes, including prolonged grief.

## Supplementary Information

Supplementary Material 1.

Supplementary Material 2.

## Data Availability

The datasets for this study are not publicly available because of the risk that individual privacy could be compromised; however, the datasets are available from the corresponding author on reasonable request.

## Declarations

### Concent for publication

Informed consent was obtained from family members by returning the questionnaires.

## Abbreviations

CCN: Critical Care Nurse
FS-ICU: Family Satisfaction with Care in the Intensive Care Unit
FS-ICU/Care: Subscale Family Satisfaction with Care
FS-ICU/DM: Subscale Family Satisfaction in Decision-making
FS-ICU/Total: Family Satisfaction in Total
ICU: Intensive Care Unit
