# Supplementary material for: Satisfaction with end-of-life care and self-rated health among bereaved family members; a descriptive cross-sectional study in an intensive care context
Source: BMC Palliat Care. 2026 Apr 30;25:125. doi: 10.1186/s12904-026-02124-x (PMC13134228; doi:10.1186/s12904-026-02124-x)
Supplement: Supplementary file 1 — Supplementary Material 1. [file 12904_2026_2124_MOESM1_ESM.docx]

**Supplemental file 1, Table 1.** Satisfaction with care from subscale FS-ICU Care showing items, response options, and response frequency in number and percentage.

| **Items** | ***n*** | ***n* (%)** | ***n* (%)** | ***n* (%)** | ***n* (%)** | ***n* (%)** |
| --- | --- | --- | --- | --- | --- | --- |
|  |  | **Excellent** | **Very good** | **Good** | **Fairly good** | **Poor** |
| How was your relative treated with regard to  **1.** courtesy, respect and compassion? | 137 | 85 (62.0) | 35 (25.6) | 10 (7.3) | 6 (4.4) | 1 (0.7) |
| **2.** pain? | 132 | 71 (53.8) | 37 (28.0) | 18 (13.6) | 3 (2.3) | 3 (2.3) |
| **3**. breathing difficulties? | 124 | 67 (54.0) | 39 (31.5) | 12 (9.7) | 5 (4.0) | 1 (0.8) |
| **4**. agitation? | 118 | 53 (44.9) | 33 (28.0) | 18 (15.3) | 7 (5.9) | 7 (5.9) |
| How were you treated by the staff with regard to  **5.** your needs? | 141 | 77 (54.6) | 35 (24.8) | 15 (10.6) | 11 (7.8) | 3 (2.1) |
| **6**. emotional support? | 138 | 71 (51.4) | 34 (24.6) | 16 (11.6) | 10 (7.2) | 7 (5.1) |
| **7**. How was the cooperation between the staff in the care of your relative? | 134 | 61 (45.5) | 39 (29.1) | 21 (15.7) | 8 (6.0) | 5 (3.7) |
| **8**. How were you treated by staff with respect to courtesy, respect and compassion? | 141 | 82 (58.2) | 40 (28.4) | 12 (8.5) | 6 (4.3) | 1 (0.7) |
| **9.** How well did the nurses care for your relative in the ICU? | 138 | 87 (63.0) | 35 (25.4) | 13 (9.4) | 3 (2.2) | 0 (0) |
|  |  | **Very often** | **Often** | **Sometimes** | **Rarely** | **Never** |
| **10.** How often did nurses talk to you about your relative’s condition? | 131 | 40 (30.5) | 61 (46.6) | 16 (12.2) | 12 (9.2) | 2 (1.5) |
|  |  | **Excellent** | **Very good** | **Good** | **Fairly good** | **Poor** |
| **11.** How well did the physicians care for your relative? | 139 | 78 (56.1) | 39 (28.1) | 13 (9.4) | 6 (4.3) | 3 (2.1) |
| **12**. How was the atmosphere in the ICU? | 138 | 55 (39.9) | 41 (29.7) | 34 (24.6) | 6 (4.3) | 2 (1.4) |
| **13**. How was the atmosphere in the relatives’ dayroom? | 105 | 26 (24.8) | 32 (30.5) | 32 (30.5) | 11 (10.4) | 4 (3.8) |
|  |  | **Very dissatisfied** | **Quite dissatisfied** | **Mostly satisfied** | **Very satisfied** | **Completely satisfied** |
| **14.** How is your total satisfaction with the care? | 141 | 5 (3.5) | 3 (2.1) | 27 (19.1) | 40 (28.4) | 66 (46.8) |
