# Supplementary material for: Satisfaction with end-of-life care and self-rated health among bereaved family members; a descriptive cross-sectional study in an intensive care context
Source: BMC Palliat Care. 2026 Apr 30;25:125. doi: 10.1186/s12904-026-02124-x (PMC13134228; doi:10.1186/s12904-026-02124-x)
Supplement: Supplementary file 2 — Supplementary Material 2. [file 12904_2026_2124_MOESM2_ESM.docx]

**Supplemental file 2, Table 2.** Satisfaction with decision-making from subscale FS-ICU Decision-making showing items, response options, and response frequency in number and percentage.

| **Items** | ***n*** | ***n* (%)** | ***n* (%)** | ***n* (%)** | ***n* (%)** | ***n* (%)** |
| --- | --- | --- | --- | --- | --- | --- |
|  |  | **Very often** | **Often** | **Sometimes** | **Rarely** | **Never** |
| **15.** How often did the physicians talk to you about your relative’s condition? | 135 | 32 (23.7) | 72 (53.3) | 16 (11.9) | 11 (8.1) | 4 (3.0) |
|  |  | **Excellent** | **Very good** | **Good** | **Fairly good** | **Poor** |
| **16.** How was the staff’s willingness to answer your questions? | 141 | 69 (48.9) | 45 (31.9) | 12 (8.5) | 12 (8.5) | 3 (2.1) |
| **17.** How well did the staff give explanations that you understood? | 138 | 65 (47.1) | 46 (33.3) | 15 (10.9) | 9 (6.5) | 3 (2.2) |
| **18**. How honest was the information you received about your relative’s condition? | 140 | 71 (50.7) | 37 (26.4) | 18 (12.9) | 8 (5.7) | 6 (4.3) |
| **19.** How well were you informed about what happened to your relative and why measures were taken? | 140 | 76 (54.3) | 0 (0) | 40 (28.6) | 13 (9.3) | 11 (7.8) |
| **20.** How consistent was the information about your relative’s condition from physicians, nurses etc. | 136 | 58 (42.6) | 34 (25.0) | 24 (17.6) | 12 (8.8) | 8 (5.9) |
|  |  | **I felt completely excluded and abandoned** | **I felt partially excluded** | **I felt neither involved nor excluded** | **I felt partially involved** | **I felt very involved** |
| **21.** Did you feel involved in the decisions that were made? | 141 | 5 (3.5) | 6 (4.3) | 24 (17.0) | 28 (19.9) | 78 (55.3) |
|  |  | **I felt that I had no control whatsoever and that the healthcare system took over and dictated the care my relative received** | **I felt that I did not have full control and that the healthcare system largely took over and dictated the care that my relative received** | **I felt neither in control nor that I didn't** | **I felt that I had some control over the care my relative received** | **I felt that I had good control over the care that my relative received** |
| **23.** Did you feel that you had control over the care your relative received? | 141 | 11 (7.8) | 13 (9.2) | 31 (22.0) | 33 (23.4) | 53 (37.6) |
|  |  | **I would have needed more time** |  |  |  | **I had enough time** |
| **24.** When decisions were to be made, did you have enough time to express your concerns and have your questions answered? | 136 | 37 (27.2) |  |  |  | 99 (72.8) |
